# Is shorter also better in the treatment of *Clostridioides difficile* infection?

**DOI:** 10.1093/jac/dkae119

**Published:** 2024-04-25

**Authors:** M Duricek, K Halmova, M Krutova, B Sykorova, J Benes

**Affiliations:** Department of Infectious Diseases, 3rd Faculty of Medicine, Charles University and University Hospital Bulovka, Budínova 67/2, 180 81, Praha 8, Prague, Czech Republic; Department of Infectious Diseases, 3rd Faculty of Medicine, Charles University and University Hospital Bulovka, Budínova 67/2, 180 81, Praha 8, Prague, Czech Republic; Department of Medical Microbiology, 2nd Faculty of Medicine, Charles University and Motol University Hospital, Prague, Czech Republic; Department of Clinical Microbiology, University Hospital Bulovka, Prague, Czech Republic; Department of Infectious Diseases, 3rd Faculty of Medicine, Charles University and University Hospital Bulovka, Budínova 67/2, 180 81, Praha 8, Prague, Czech Republic

## Abstract

**Objectives:**

To assess the effectiveness of shortened regimens of vancomycin or fidaxomicin in the treatment of *Clostridioides difficile* infection (CDI).

**Methods:**

Adult patients with CDI hospitalized from January 2022 to May 2023 were included in this observational study. In patients with CDI treated with vancomycin or fidaxomicin, antibiotic treatment was discontinued after either 5 or 7 days of vancomycin or 5 days of fidaxomicin if there was a clinical response and improvement in laboratory parameters. The control cohort was treated with the standard 10 day regimen of either vancomycin or fidaxomicin. The follow-up was 60 days. Causative *C. difficile* strains were characterized by ribotyping and toxin gene detection when available.

**Results:**

Twenty-five patients (median age 76 years) received shortened treatment with vancomycin (*n* = 21), or fidaxomicin (*n* = 4). Five cases fulfilled the criteria for severe CDI. Twenty-three patients completed follow-up; two died from causes other than CDI, and two developed recurrent CDI (8.0%). Ribotypes (RTs) 001 and 014 were the most prevalent with 20% each. In two *C. difficile* isolates, binary toxin genes were detected (RTs 078 and 023). In the control group of 22 patients recurrent CDI developed in 5 patients (22.7%). No statistically significant differences were found between the groups.

**Conclusions:**

Shortened treatment regimens for CDI with vancomycin and fidaxomicin were shown to be effective in our cohort of patients compared with 10 days of treatment. The recurrence rate was lower in the study group. A larger, prospective, double-blind, randomized, multicentre study is needed to support our findings.

## Introduction

Since their discovery in the 20th century antibiotics have provided highly effective treatment of bacterial infectious diseases.^1^ But besides their beneficial effects, adverse events like dysbiosis can emerge during or after the antimicrobial treatment. This imbalance of the bacterial ecosystem is caused by a reduction of species diversity, which impairs natural colonization resistance, creating an opportunity for *Clostridioides difficile* to adhere, multiply and produce toxins, which leads to inflammation and subsequently to diarrhoea, which can be complicated with dehydration and deterioration of the patient’s clinical condition.^2,3^ Furthermore, 15%–35% of patients develop recurrent *C. difficile* infection (rCDI).^4^

Currently, fidaxomicin and vancomycin are recommended in CDI treatment, fidaxomicin as a drug of first choice with vancomycin as an alternative.^5^ Historically, metronidazole for 10 days was used as the first-choice treatment.^6^ Therefore, the duration of CDI treatment remained at 10 days in all subsequent clinical trials that assessed its effectiveness.

In the last few decades, the use of antibiotics has been reconsidered, and antimicrobial stewardship (AMS) programmes have been established. The basic goals of AMS are the appropriate use of antibiotics with a positive impact on patient care, a decrease in antibiotic resistance and a reduction of medical expenses.^7^ The length of antibiotic therapy has been reconsidered in pharyngitis, pneumonia, urinary tract infections and other bacterial infections.^8–10^

Both the European and US CDI treatment guidelines recommend prolonged antibiotic therapy (taper or taper and pulse regimen), which reduces the risk of rCDI at the cost of patient comfort, increased treatment costs, and selection of resistant bacteria in the patient’s gastrointestinal tract.^5,11^ However, after just 3 days of oral vancomycin or 1–2 days of fidaxomicin stool concentrations of both fidaxomicin and vancomycin can be hundreds-fold to even thousands-fold MICs, and the clinical resolution of diarrhoea is achieved, in some patients.^12,13^

To assess whether shortened CDI-specific treatment regimens are effective in patients with clinical response, an observational pilot study was conducted. To the best of our knowledge, our study presents the first clinical data for a shortened treatment for CDI.

## Materials and methods

This observational study was performed from January 2022 to May 2023 at the Department of Infectious Diseases of University Hospital Bulovka, Prague. Adult patients with CDI were treated with a standard monotherapy of vancomycin 125 mg q6h or fidaxomicin 200 mg q12h. A good clinical and laboratory treatment response allowed for the shortening of the antibiotic regimen in compliance with the Czech national guidelines.^13^ The observed laboratory parameters were WBC count and C-reactive protein (CRP) serum levels. The control group of patients treated with the standard 10 day regimen of either vancomycin or fidaxomicin was included.

Patient characteristics were compared using the Fisher exact test for categorical variables and the exact Wilcoxon–Mann–Whitney test for continuous variables.

### Ethical approval

The study was approved by the University Hospital Bulovka Ethics Committee in 2022 (evidence number 10648EK-Z).

### Laboratory diagnostics of CDI

Unformed stool samples were tested for the presence of *C. difficile* glutamate dehydrogenase (GDH) and toxins A/B by enzyme immunoassay (C. DIFF QUIK CHEK COMPLETE^™^; TECHLAB, USA). In case of a positive GDH result without toxin A/B detection and a clinical picture of CDI with negative detection of other intestinal pathogens, the presence of toxin genes was detected by a nucleic acid amplification test (NAAT) (Xpert C. difficile BT; Cepheid AB, Sweden) or toxigenic *C. difficile* culture was performed. Ribotyping was performed on the causative *C. difficile* strains.^14^

### Assessment of the recurrence risk

All patients were evaluated for the presence of risk factors for CDI recurrence. Risk factors listed in the current European guidelines were used: an older age of the patient (>65 years); healthcare-associated CDI; prior hospitalization in the last 3 months; use of concomitant antibiotics; protein pump inhibitors started during/after CDI diagnosis; and prior CDI episode.^5^ Recurrence risk was calculated as the number of these factors present in each patient’s history [see Tables 1 and 2 and Table S1 (available as Supplementary data at *JAC* Online)].

**Table 1.** Comparison of study and control groups^a^

	Shortened treatment regimen (*n* = 25)	Standard treatment regimen (*n* = 22)	*P* value
*Patient characteristics*			
Gender (%)			0.58
Male	11 (44.0)	7 (31.8)	
Female	14 (56.0)	15 (68.2)	
Age, median, y (range)	76 (66.0–83.0)	74.5 (73.0–82.8)	0.20
Recurrence risk (mean)	3.3 (1.2)	2.4 (1.2)	
*CDI treatment (%)*			0.79
Vancomycin	21 (84.0)	20 (90.9)	
Fidaxomicin	4 (16.0)	2 (9.1)	
*Blood parameters*			
Leucocyte count at the beginning, cells/10^9^/L	10.0 (6.2–13.2)	10.8 (8.6–14.5)^b^	0.15
Leucocyte count at the end, cells/10^9^/L	7.5 (6.5–9.1)^b^	7.0 (5.6–9.4)^b^	0/76
C-reactive protein at the beginning, mg/L	78.3 (42.5–115.7)	49.9 (30.9–85.4)^b^	0.93
C-reactive protein at the end, mg/L	30.4 (13.2–54.9)^b^	11.0 (6.8–37.1)^b^	0.92
*CDI characteristics and outcomes (%)*			
First episode at the hospital admission	22 (88.0)	17 (77.3)	NA
Recurrent CDI at the hospital admission	3 (12.0)	3 (13.6)	
Other than first CDI episode at the hospital admission	0 (0.0)	2 (9.1)	
Recurrent CDI during follow-up	2 (8.0)	5 (22.7)	0.24

NA, not available.

^a^Data are *n* (%), median (IQR) or mean (SD).

^b^Missing data in the dataset.

**Table 2.** Study group data overview^a^

Patient characteristics				Diagnostics					Blood parameters				
Patient	Gender	Age	Recurrence risk	Detection of toxin in stool sample	Additional examination (NAAT or microbiology culture) from stool sample	Ribotype	Toxin genes	Therapeutic regimen	WBC at the beginning of the treatment (× 10^6^/L)	WBC at the end of the treatment (× 10^6^/L)	CRP at the beginning of the treatment (mg/L)	CRP at the end of the treatment (mg/L)	Follow-up
1	M	88	4	Positive	MC positive	014	A, B	Vancomycin (5 days)	11.8	9.1	114.2	123.7	Sustained cure
2	M	83	4	Positive		017	A, B		11.0	12.6	70.2	61	
**3**	**F**	**85**	**4**	**Positive**		**014**	**A, B**		**15**.**1**	**8**.**4**	**102**.**6**	**54**.**1**	
4	F	58	3	Negative	NAAT positive	NA	NA		7.8	6.8	97	9.8	
5	F	77	4	Positive	MC positive	001	A, B		11.9	7.5	78.3	27.1	rCDI
6	F	52	4	Positive	NP	NA	NA		13.2	9.8	60	8.9	Sustained cure
7	M	79	2	Positive	MC positive	001	A, B		10.0	NA	197.6	50.4	
8	M	69	1	Positive		002	A, B		6.5	6.5	35.3	NA	rCDI
**9**	**F**	**88**	**4**	**Positive**	NP	**NA**	**NA**		**18**.**7**	**6**.**7**	**12**.**1**	**8**.**3**	**Sustained cure**
**10**	**F**	**74**	**5**	**Positive**	MC positive	**001**	**A, B**		**20**.**5**	**8**.**8**	**294**.**9**	**58**.**8**	
11	M	36	1	Positive		023	A, B, Binary		8.9	6.2	161.2	77.1	Sustained cure
12	M	87	4	Positive	NAAT positive	NA	NA		5.4	NA	9.9	NA	
13	F	84	4	Positive	MC positive	014	A, B		6.2	NA	62.6	20.5	
14	F	76	3	Negative	NAAT positive	012	A, B		1.2	6.6	42.5	34.9	
15	M	34	1	Positive	MC positive	078	A, B, Binary		1.6	2.7	115.7	36.4	
**16**	**F**	**27**	**1**	**Negative**	**NAAT positive**	**015**	**A, B**		**17**.**3**	**11**.**2**	**138**.**5**	**55**.**7**	**Sustained cure**
17	M	82	4	Positive	MC positive	106	A, B	Vancomycin (7 days)	14.6	10.7	59.2	50.8	Death
18	F	86	3	Negative	NAAT positive	001	A, B		9.6	6.5	54.5	16.5	Sustained cure
19	M	72	4	Negative		013	A, B		9.5	6.8	192	26.3	
20	F	61	3	Negative	MC positive	005	A, B		10.6	8.7	34	7.8	
21	M	76	4	Positive		029	A, B		4.5	3.5	16.2	8.5	
22	F	72	4	Positive		014	A, B	Fidaxomicin (5 days)	0.8	6	33.2	26.9	
**23**	**F**	**76**	**2**	**Positive**		**043**	**A, B**		**21**.**9**	**9**.**1**	**81**.**5**	**30**.**4**	**Sustained cure**
24	M	72	5	Negative		013	A, B		6.0	9.5	222.8	70.4	Death
25	F	66	4	Negative	NAAT positive	NA	NA		10.4	NA	78.7	4.4	Sustained cure

A, toxin gene A (*tcdA*); B, toxin gene B (*tcdB*); Binary, binary toxin genes (*cdtA* and *cdtB*); CRP, C-reactive protein; F, female; M, male; MC, microbiology culture; NA, not available; NAAT, nucleic acid amplification test; NP, not performed; rCDI, recurrent *Clostridioides difficile* infection.

^a^Severe CDI cases are depicted in bold.

### Evaluation of treatment success

All patients were hospitalized throughout the entire course of the CDI treatment, and they were examined daily by the attending physician, who assessed their current health status. Treatment response was evaluated according to clinical symptoms, such as the resolution of diarrhoea and abdominal pain.^5^ In most patients, inflammatory markers—WBC count and serum CRP level—were examined at the beginning and end of CDI treatment.

### Follow-up

Upon discharge, all patients were advised to attend for examination in case of disease recurrence. Additionally, they were checked during an outpatient or telephone inspection 2 months after the illness ended, which is a part of standard care for all patients hospitalized with a diagnosis of CDI. If patients could not be contacted directly, their relatives or their attending physicians were interviewed.

## Results

### CDI patients with a shortened regimen (study group)

In total, 25 patients fulfilled the study criteria and continued with additional follow-up. Of the 25 patients enrolled in the study group, 22 patients (88%) had their first CDI episode and 3 patients suffered from rCDI at hospital admission. Five patients (20%) had a severe course of CDI due to leucocytosis >15.0 × 10^9^/L at presentation. The patients’ ages ranged between 27 and 88 years, with an average of 70 years and a median of 76 years. The majority of patients were females (56%).

All stool samples were positive for GDH, and toxins were detected in 17 cases (68%). PCR was performed on six stools with a GDH-only positive result; in these stools, the gene for toxin B (*tcdB*) was detected.

*C. difficile* ribotypes (RTs) were obtained from 20 isolates (80%); RTs 001 and 014 were the most prevalent, each accounting for 20%. Furthermore, two *C. difficile* isolates carried binary toxin genes (RTs 078 and 023). In patients with severe CDI, four different RTs were detected (001, 014, 015 and 043).

Vancomycin was administered for 5 days in 16 patients (64%) and for 7 days in 5 patients (20%). Four patients (16%) received fidaxomicin for 5 days.

Of the 25 patients, 2 patients (8%) died during follow-up at Days 6 and 16, and 2 patients had a recurrence (8%) at Days 24 and 38.

### CDI patients with the standard regimen (control group)

Twenty-two patients were included. The control group had an age range of 53–92 years, with an average age of 76 years and a median age of 74.5 years, and 68.2% of the group were females. Three patients (13.6%) had rCDI at hospital admission. The severe course of CDI was diagnosed in five patients (22.7%).

Stool samples were all positive for GDH and toxin was detected in 18 cases (82%). PCR, however, was performed in only two GDH-only positive results, with toxin B positivity in both cases.

The *C. difficile* RTs were obtained from nine isolates (40.9%). RT 014 was the most prevalent (33.3%).

The majority of patients were treated with vancomycin (90.9%) and two patients received fidaxomicin (9.1%). Recurrence of CDI was observed in five patients (22.7%).

### CDI patient groups comparison

Statistical analysis of the control and study group showed no statistically significant difference in patient characteristics such as age, gender, rCDI, WBC and CRP between the two groups.

The data for the comparison between the study group and control group are presented in Table 1, and a summary of patient data is provided in Tables 2 and Table S1.

## Discussion

CDI, especially rCDI, represents a burden for patients, hospitals and society. Current therapeutic strategies in CDI are primarily focused on clinical improvement by the decrease of *C. difficile* cells capable of toxin production, which is usually achieved with recommended antibiotic therapy for 10 days. For patients with rCDI, prolonged treatment with taper and pulse dosage has been recommended.^5^ However, this strategy contradicts AMS principles.

Dysbiosis is the essential underlying pathogenetic condition for CDI development. Vancomycin has a broad antimicrobial activity spectrum compared with fidaxomicin. Therefore, the oral administration of vancomycin can cause intestinal dysbiosis or exacerbate pre-existing dysbiosis.^15,16^

Studies of the pharmacological properties of vancomycin and fidaxomicin show that stool concentrations of these two antibiotics achieve hundreds- to even thousands-fold MICs, and resolution of diarrhoea can be achieved within 3 days with vancomycin and in 1–2 days with fidaxomicin.^12,16–18^ So, it is the early phase of the CDI treatment that reduces active *C. difficile* cells capable of toxin production. Therefore, further continuation of CDI antibiotic therapy might be of little benefit and, on the contrary, can cause exacerbation of dysbiosis with all the possible negative consequences. Our data support this hypothesis.

In 25 patients in our study group, the treatment of CDI was discontinued due to a good clinical and laboratory response at Day 5 or 7 of the treatment. Two patients died due to advanced comorbidities. Two patients developed a recurrence, and the remaining 21 patients had a sustained clinical response without a recurrence for 60 days after the end of the treatment. The majority of patients were more than 65 years old (76%).

Current studies estimate the development of rCDI in approximately 15%–35% of CDI patients; however, these estimations differ greatly.^4,19^ A recently published pan-European multicentre cost and resource utilization study recorded a recurrence rate of 18%.^20^ In our study, the recurrence rate in patients with completed follow-up in the study group was 8.0%. However, the control group experienced a higher percentage of recurrence development despite having a lower recurrence risk (mean 2.4 versus 3.3, respectively) at 22.7%. Conversely, the study group had a higher percentage of patients experiencing their first CDI episode at 88%, compared with 77% in the control group. This suggests lower rCDI rates with the shortened regimen. Undoubtedly, these results need to be confirmed in a larger cohort.

Five patients with severe CDI were identified in both the study group and the control group. However, the severity was based only on a leucocyte count >15.0 × 10^9^/L. All study group patients achieved treatment response and no recurrence occurred during the follow-up in these severe CDI cases. In the control group, one patient developed rCDI after a severe CDI episode.

The spectrum of detected RTs in our study was diverse and similar to types reported in the Czech Republic.^21^ The epidemic RT 001 was detected in 20% of patients in the study group, and RT 027 and its genetically related RT 176 were not detected. These findings are likely a result of the evolving epidemiological situation in the Czech Republic. Furthermore, the distribution of RTs may vary depending on the healthcare facilities, as was demonstrated in a comparison of RT proportions among the different hospitals in the Czech Republic.^22,23^

### Conclusion

Our data show that shortened antibiotic therapy of CDI could be easier, cheaper, less bothersome and even less risky than the current standard. Our pilot study shows promising results for a sustained clinical cure in CDI patients treated with a shorter regimen with vancomycin or fidaxomicin. A double-blind, randomized, placebo-controlled trial is needed to support our results.

## Supplementary Material

dkae119_Supplementary_Data

## Supplementary data

Table S1 is available as Supplementary data at *JAC* Online.
