# Supplementary material for: Is shorter also better in the treatment of Clostridioides difficile infection?
Source: J Antimicrob Chemother. 2024 Apr 25;79(6):1413–7. doi: 10.1093/jac/dkae119 (PMC11144488; doi:10.1093/jac/dkae119)
Supplement: dkae119_Supplementary_Data [file dkae119_supplementary_data.docx]

Table S1 Control group data overview.

| **Patient characteristics** | | | | **Diagnostics** | | | | **Therapeutical regimen** | **Blood parameters** | | | | **Follow-up** |
| --- | --- | --- | --- | --- | --- | --- | --- | --- | --- | --- | --- | --- | --- |
| **Patient** | **Gender** | **Age** | **Recurrence risk** | **Detection of toxin in stool sample** | **Additional examination**  **(NAAT or microbiology culture) from stool sample** | **Ribotype** | **Toxin genes** |  | **WBC at the beginning of the treatment**  **(x 10^6^/L)** | **WBC at the end of the treatment**  **(x 10^6^/L)** | **CRP at the beginning of the treatment**  **(mg/L)** | **CRP at the end of the treatment**  **(mg/L)** |  |
| 1 | F | 92 | 5 | positive | NP | NA | NA | Vancomycin  (10 days) | 7.2 | 5.9 | 79.5 | NA | rCDI |
| 2 | F | 89 | 3 | negative | NAAT positive |  |  |  | 10.6 | 4.8 | 71.4 | 10.9 |  |
| 3 | M | 76 | 1 | negative |  | 014 | A, B |  | 8.6 | 8.2 | 4.9 | 6.4 |  |
| 4 | M | 75 | 3 | positive |  | 001 | A, B |  | 8.8 | NA | 6.9 | NA |  |
| **5** | **F** | **89** | **2** | **positive** | **MC positive** | **AI-75** | **A, B** |  | **19.9** | **9.8** | **105.6** | **11.2** | **rCDI** |
| **6** | **F** | **78** | **4** | **positive** | **NP** | **NA** | **NA** |  | **15.1** | **14.4** | **218.1** | **114.8** | **Sustained cure** |
| 7 | M | 74 | 3 | positive | NP | NA | NA |  | NA | NA | NA | NA | Sustained cure |
| 8 | F | 74 | 2 | positive |  |  |  |  | 9.2 | NA | 85.4 | NA |  |
| 9 | M | 73 | 3 | positive |  |  |  |  | 12.1 | 11.3 | 217.3 | 228.5 |  |
| 10 | F | 74 | 3 | positive |  |  |  |  | 6.6 | 5.2 | 30.9 | 37.1 |  |
| 11 | F | 71 | 3 | positive |  |  |  |  | 8.0 | NA | 18.7 | NA |  |
| 12 | M | 83 | 3 | positive |  |  |  |  | 12.6 | 5.9 | 33.0 | 6.8 |  |
| 13 | F | 78 | 2 | negative | MC positive | NA | NA |  | 5.0 | NA | 106.4 | NA |  |
| 14 | M | 82 | 2 | positive |  | 014 | A, B |  | 13.8 | 9.8 | 32.2 | 6.8 |  |
| 15 | F | 92 | 1 | positive |  | NA | NA |  | 9.0 | NA | 2.5 | NA |  |
| 16 | F | 73 | 3 | positive |  | 014 | A, B |  | 13.7 | 3.8 | 41.3 | 3.9 |  |
| 17 | M | 62 | 0 | positive |  | 070 | A, B |  | 6.9 | NA | 44.5 | NA |  |
| 18 | F | 59 | 0 | positive |  | 412 | A, B |  | 10.8 | NA | 60.8 | NA |  |
| 19 | F | 91 | 1 | positive |  | 001 | A, B |  | 14.5 | 7.3 | 28.0 | 63.2 |  |
| **20** | **F** | **65** | **2** | **positive** | **NP** | **NA** | **NA** |  | **17.3** | **6.7** | **49.9** | **3.1** | **Sustained cure** |
| **21** | **F** | **73** | **3** | **positive** | **NP** | **002/2** | A, B | **Fidaxomicin (10 days)** | **17.4** | **5.5** | **66.5** | **14.3** | **Sustained cure** |
| **22** | **F** | **53** | **3** | **negative** | **NAAT positive** | **NA** | **NA** |  | **24.7** | **7.4** | **118.0** | **11.0** |  |

M – male; F – female; NP – not performed; NA – not available; MC – microbiology culture; WBC – white blood cells; CRP – C-reactive protein; severe CDI cases are depicted in bold.
